# Headache in the Neurological Emergency Department—High Degree of Inadequate Documentation Calls for Structured Assessments

**DOI:** 10.3389/fneur.2022.847484

**Published:** 2022-03-24

**Authors:** Florian Rimmele, Josephine Janke, Peter Kropp, Ramon Kasch, Uwe Walter, Tim P. Jürgens

**Affiliations:** ^1^Department of Neurology, Headache Center North-East, University Medical Center Rostock, Rostock, Germany; ^2^Institute of Medical Psychology and Medical Sociology, University Medical Center Rostock, Rostock, Germany; ^3^Department of Neurology, KMG Klinikum Güstrow, Güstrow, Germany

**Keywords:** emergency department (ED), headache, migraine, red flags, (ICHD-3) International Classification of Headache Disorders, third edition, structured assessment, documentation

## Abstract

Headaches are a frequent reason for presentation to the emergency department (ED) and can pose a great challenge for the attending physicians. First and foremost, the distinction between a primary and secondary headache with potentially life-threatening implications can be difficult. Moreover, it often occurs that no specific headache diagnosis is made at discharge from the ED. Therefore, in this present retrospective cross-sectional study, all patients who presented to the emergency department of the Department of Neurology at Rostock University Medical Centre with the main symptom of headache between November 2013 and November 2016 underwent a neurological examination and the extent to which warning symptoms (“red flags”) for a secondary headache as well as symptoms necessary for a correct headache diagnosis according to the ICHD-3 classification were recorded and documented. We could show that documentation of red flags and clinical characteristics is inadequate and does not allow proper diagnostic categorization. To facilitate concise documentation and rapid decision making we suggest a structured and standardized form for documenting the headache history and red flags in the ED.

## Introduction

Headaches are a frequent reason for presentation to the emergency department (ED), especially among neurological patients (8.4–16%) (1, 2). While primary headaches such as migraine are predominant (3), there is an inherent risk that secondary headaches with potential life-threatening implications illness remain undetected in the ED (4). In a retrospective cross-sectional study carried out by our group in patients with the cardinal symptom of headache in a neurological emergency department, we had previously found a headache prevalence of 9.1% (5). Of these, primary headaches amounted to 40.1%, while secondary headaches were found in 29%. To support the ED physician in distinguishing between primary and secondary headache, national guidelines and the concept of warning symptoms (“red flags”) have been published (4, 6–8). Once a secondary headache has been ruled out, the correct diagnosis of the headache type based on the ICHD-3 classification (9) allows initiation of the appropriate treatment (3). Primary headache is frequently under- or misdiagnosed in the ED, resulting in inadequate treatment (10–12). Indeed, we found that 24.7% of patients with headache in the neurological ED did not receive a specific diagnosis beyond headache as such (5). Therefore, the present study aimed to enquire how often warning symptoms (“red flags”) were documented and which essential clinical characteristics allowing diagnosis according to the ICHD-3 criteria were recorded.

## Methods

We conducted a retrospective analysis based on the data recorded in the previously published study from our group on patients who presented to the neurological ED at Rostock University Medical Center with the main symptom of headache between November 2013 and November 2016 (5). In detail, it was recorded whether warning symptoms (“red flags”) for a secondary headache, as well as core clinical characteristics essential for correct headache diagnosis using the ICHD-3 classification were documented in the medical charts. The data were extracted from the digital doctor's notes in the clinical information system. Descriptive statistical analyses were done with Excel (MS Office Professional Plus 2016). Categorical data were reported in numbers and percentages. Red flags were defined according to expert opinion and national guidelines (6, 7, 13–16), the characteristics relevant for headache diagnosis in line with the ICHD-3 criteria (9). The research was conducted according to the Declaration of Helsinki. The retrospective, fully anonymized analysis of data was approved by the Ethics committee of the University Medical Center Rostock (identifier: A 2018/0168).

## Results

The study included 11,210 patients who presented to the neurological ED of the University Medical Center Rostock between November 2013 and November 2016. Of these, 1,020 suffered from headache as their cardinal symptom (9.1%).

### Red Flags

No doctors' note contained all red flags identified by our group. Surprisingly, the presence or absence of thunderclap headache was not recorded. Other items were recorded and documented much more frequently, such as “recent headache or change in headache pattern” (49.8%) or also “fever” and “age,” which was systematically documented as part of the admission routine (Table 1).

**Table 1:** List of documented red flags (warning signs and symptoms for secondary headache) for emergency headache patients.

**Red flags**	**Number collected and documented (%)**
1. Age above 50	1,020 (100)
2. Neoplasm in history	45 (4.4)
3. Immunosuppression in history^*^	16 (1.6)
4. Cardiovascular diseases in history	234 (22.9)
5. Posttraumatic onset of headache	37 (3.6)
6. Thunderclap headache	Not reported
7. Recent onset or pattern change of headache	508 (49.8)
8. Positional headache (Valsalva, cough, sneeze)	32 (3.1)
9. Fever	1,020 (100)
Red flag 1 to 9	0 (0)

* *Recorded as rheumatological/autoimmune disease in history*.

### Headache Characteristics

A complete set of headache characteristics essential for ICHD-3 diagnosis was not recorded in any data set (*n* = 0). When excluding the item “headache localization” which was not reported in any note, a full set of residual characteristics was documented in 22.7% (*n* = 232). On an individual level, items such as “headache duration” (68.4%), “pain intensity” (43.2%), and “headache character” (42.1%) were documented more frequently (Table 2).

**Table 2:** List of documented signs or symptoms for diagnosis according to ICHD-3 classification for emergency headache patients.

**Signs or symptoms**	**Number collected and documented (%)**
1. History of primary headache (or similar episodes)^*^	171 (16.8)
2. Headache localization (one-sided, two-sided)	Not reported
3. Headache character (dull, pulsating)	429 (42.1)
4. Pain intensity	441 (43.2)
5. Headache duration	698 (68.4)
6. Nausea	343 (33.6)
7. Photo-, Phonophobia	168 (16.5)
8. Accompanying autonomic symptoms	28 (2.7)
Signs or symptoms 1 to 8 (without 2.)^**^	232 (22.7)

* *Only migraine reported in history, no other primary or secondary headaches*.

** *The sum score presented in the last line corresponds to the number of patients for whom all points (1 to 8 without 2) were recorded*.

## Discussion

Headaches are a common complaint in the population (17) and a frequent reason for presentation to an ED (18). The majority of headaches in patients in the ED are attributable to primary headache such as migraine or others with a benign origin. The physicians' crucial task is to identify those with a potentially life-threatening secondary cause (3, 4). Several instruments to support physician's decision-making based on the presence of warning sign or symptoms (“red flags”) have been proposed (6, 7, 19). In the present study, we examined the extent to which these were actually used in the daily routine of the ED based on doctors' notes. We defined a standard set of red flags (Table 1) based on the SNNOOP10 list and national and regional guidelines (19). The complete set was not recorded in one patient. This corresponds to the results of Locker et al. (1), who found that a complete headache history was only documented in 0.3% of cases (1 out of 353). Nevertheless, in our study, it emerged that individual points were recorded more frequently, such as “Recent onset or pattern change of headache” at 49.8%. Individual items such as neoplasia in the medical history were documented if present in the residual notes. This illustrates the dilemma of missing documentation in unstructured interviews and absence of check lists. It can either be due to the fact that patients were not asked about the presence of characteristics or due to the fact that the corresponding characteristics was not present and therefore not recorded. It is nevertheless striking and disconcerting that some essential aspects such as the presence or absence of thunderclap headache were not documented at all. Another example is the age of the patient, which was automatically documented. However, this does not necessarily mean that the attending physician perceived an age over 50 years as a “red flag” in headache patients and noted it accordingly.

We further evaluated the extent to which characteristics and symptoms important for reaching the correct diagnosis were recorded in our study. A high number of patients (24.7%) were discharged without a specific headache diagnosis. This is a relatively small proportion compared to other studies, in which as many as 33.9% (20) and up to 59% of patients (11) did not receive a specific headache diagnosis. It may be due to the fact that all patients in our study were seen by a neurologist instead of emergency physicians. As for red flags, we defined a standard set of characteristics important for making a diagnosis according to the ICHD-3 criteria (8) (Table 2). Again, we found that the full list of items was not documented for any patient. Nevertheless, individual items were documented substantially more often (Table 2).

A decisive reason for the high number of patients discharged without a specific headache diagnosis is that physicians in the ED only rarely use the ICHD-3 criteria for headache diagnosis, as was also described by Granato et al. (20). The potential reasons for this are diverse. In a setting with limited time resources, the detection of life-threatening conditions is more important than a correct ICHD-3 diagnosis based on a comprehensive headache interview. In addition, inexperienced clinicians may not be familiar with the ICHD-3 classification. However, a systematic assessment of key headache characteristics can additionally help stratifying patients using positive predictors for primary headache (“green flags”) together with warning symptoms (“red flags”). Munoz-Ceron et al. (4) found that the fulfillment of the ICHD-3 criteria was the best predictor of primary headache.

A specific diagnosis at discharge is crucial for appropriate treatment in the ED and also for adequate subsequent post ED treatment (3, 20). A standardized documentation across different hospitals could facilitate research and clinical audits as suggested by Locker et al. (1).

Finally, inadequate documentation of red flags and clinical characteristics can have serious legal implications. In the German medicolegal system, information that was collected in a medical interview and not documented in the notes is considered not to have been gathered at all. Therefore, checklists and structured interviews should be mandatory in the ED and ideally included as decision making tool in the clinical information system.

Therefore, we suggest to record warning signs and symptoms in a short, structured, and standardized form as part of the headache history. This approach has already led to substantial improvements in other areas of emergency medicine (21). The structured headache interview suggested by us is short, concise, and feasible for use in acute medical settings with time constraints (Table 3). It includes warning symptoms for secondary headache (“red flags”) as described in the literature (13, 19) and additionally, “cardiovascular diseases in history,” for which we found a high correlation with secondary headache (5). Furthermore, it addresses characteristics and symptoms important for the diagnosis of a primary headache according to the ICHD-3 classification (8). We have implemented this standardized headache history form in the digital patient record documentation system and will assess quality of documentation. Lastly, we included “green flags” as recently suggested by an expert panel identified in a Delphi process (22). Helpful tools for evidence-based decision making for headache diagnosis in the ED as proposed by Cortelli et al. are an important tool for improving the care of headache patients in the ED (23). Structured recording of headache characteristics is also critical for this purpose. Using a structured tool can simplify and speed up decision-making on diagnosis and treatment of potentially life-threatening headaches, while ensuring adequate documentation. This decision support can be particularly helpful in non-neurological settings and physicians with little clinical experience. The rapid identification of potentially perilous headaches in the notoriously busy ED with limited temporal resources should be followed by sufficient and specialized further care of the patient in the outpatient sector, ideally integrated into a regional network including the ED as well.

**Table 3:** Structured headache history: core headache characteristics, red and green flags.

	**Yes**	**No**
**Headache characterization**		
1. History of primary headache		
2. Similar episodes		
If yes, then is the number >5		
3. Headache character:		
Dull		
Pulsating		
Other		
4. Pain intensity (VAS or NRS)	/10	
5. Headache duration:		
Sec.		
Hours-days		
Permanent		
Other		
6. Nausea		
7. Photo-, Phonophobia		
8. Accompanying autonomic symptoms		
**Red flags**		
9. Age over 50 years		
10. Neoplasm in history		
11. Immunosuppression in history		
12. Cardiovascular diseases in history		
13. Posttraumatic onset of headache		
14. Thunderclap onset		
15. Recent onset or changing headache pattern		
16. Alarming headache triggers: positional change, Valsalva, cough, sneeze		
17. Medication: Anticoagulants		
Painkillers overuse		
18. Fever		
**Green flags**		
19. Headache has already been present in childhood		
20. Presence of headache free days		
21. Headache occurs in close temporal relationship with menstrual cycle		
22. Close family members have the same headache phenotype		
23. Headache occurred or stopped more than a week ago		

*Green flags were cited from (22). NRS, numeric rating scale; VAS, visual analogue scale*.

## Limitations

The study has several potential limitations that need to be addressed. It was a retrospective cross-sectional data analysis, and all clinical data were collected by the attending physician and documented in the patient record. This procedure carries the risk of errors when recording the data. A systematic selection bias is also possible, but unlikely, since the individual data records have been collected by many different doctors over a long period. For the data collection, there was no uniform and standardized questionnaire or procedure. From this study, it is only possible to make statements about how often the individual items were documented. It may well be that individual warning symptoms (“red flags”) were asked about and were included in further decision-making on diagnosis, therapy, or even arriving at a final diagnosis without being explicitly documented.

## Data Availability Statement

The original contributions presented in the study are included in the article/supplementary material, further inquiries can be directed to the corresponding author.

## Ethics Statement

The studies involving human participants were reviewed and approved by Ethics Committee of the University Medical Center Rostock (A 2018/0168). Written informed consent for participation was not required for this study in accordance with the national legislation and the institutional requirements.

## Author Contributions

FR: drafted the manuscript, analysis and interpretation, and revising the manuscript. JJ: acquisition, analysis, interpretation, and revising the manuscript. PK: analysis, interpretation, and revising the manuscript. RK and UW: interpretation and revising the manuscript. TJ: conception, analysis, interpretation, and revising the manuscript. All authors contributed to the article and approved the submitted version.

## Conflict of Interest

FR served on advisory boards and/or as speaker for Allergan, Novartis, Teva, Ipsen, Lilly. He has received royalties from Elsevier. PK served on advisory boards and/or as speaker for Allergan, Novartis, Teva, and Lilly. UW has received speaker honoraria and travel funds from Ipsen Pharma, Merz Pharma, Allergan, Bristol-Myers Squibb, Daiichi Sankyo, Bayer Vital, Boehringer Ingelheim and Pfizer, and a research grant from Merz Pharma. He has received royalties from Thieme and Elsevier Press. He serves as editor of the European Journal of Ultrasound. TJ served on advisory boards and/or as speaker for Allergan, Autonomic Technologies, Desitin, Hormosan, Novartis, Lilly, Lundbeck, Sanofi, and Teva. The remaining authors declare that the research was conducted in the absence of any commercial or financial relationships that could be construed as a potential conflict of interest.

## Publisher's Note

All claims expressed in this article are solely those of the authors and do not necessarily represent those of their affiliated organizations, or those of the publisher, the editors and the reviewers. Any product that may be evaluated in this article, or claim that may be made by its manufacturer, is not guaranteed or endorsed by the publisher.

## References

[1] Locker T Mason S Rigby A. Headache management–are we doing enough? An observational study of patients presenting with headache to the emergency department. Emerg Med J. (2004) 21:327–32. 10.1136/emj.2003.012351 15107372 PMC1726346

[2] Schankin CJ Straube A Bassetti CL Fischer U. Headache in the emergency department. Nervenarzt. (2017) 88:597–606. 10.1007/s00115-017-0335-x 28466105

[3] Giamberardino MA Affaitati G Costantini R Guglielmetti M Martelletti P. Acute headache management in emergency department. A narrative review. Intern Emerg Med. (2020) 15:109–17. 10.1007/s11739-019-02266-2 31893348

[4] Munoz-Ceron J Marin-Careaga V Peña L Mutis J Ortiz G. Headache at the emergency room: etiologies, diagnostic usefulness of the ICHD 3 criteria, red and green flags. PLoS ONE. (2019) 14:e0208728. 10.1371/journal.pone.0208728 30615622 PMC6322863

[5] Rimmele F Janke J Kropp P Grossmann A Hamann T Walter U. Headache characteristics in the neurological emergency department: a retrospective study. Front Neurol. (2021) 12:706074. 10.3389/fneur.2021.706074 34489852 PMC8416997

[6] Peretz A Dujari S Cowan R Minen M. ACEP guidelines on acute nontraumatic headache diagnosis and management in the emergency department, commentary on behalf of the refractory, inpatient, emergency care section of the American Headache Society. Headache. (2020) 60:643–6. 10.1111/head.13744 31944291 PMC8640610

[7] Diagnostik und apparative Zusatzuntersuchungen bei Kopfschmerzen (2012). Available online at: https://www.dmkg.de/files/dmkg.de/Empfehlungen/Leitlinie_Diagnostik_Zusatzuntersuchungen_bei_Kopfschmerzen_2017.pdf

[8] Mitsikostas DD Ashina M Craven A Diener HC Goadsby PJ Ferrari MD. European Headache Federation consensus on technical investigation for primary headache disorders. J Headache Pain. (2015) 17:5. 10.1186/s10194-016-0596-y 26857820 PMC4747925

[9] Headache Classification Committee of the International Headache Society (IHS). The International Classification of Headache Disorders, 3rd edition. Cephalalgia. (2018) 38:1–211. 10.1177/0333102417738202 29368949

[10] Valade D Lucas C Calvel L Plaisance P Derouet N Meric G. Migraine diagnosis and management in general emergency departments in France. Cephalalgia. (2011) 31:471–80. 10.1177/0333102410378178 20670996

[11] Blumenthal HJ Weisz MA Kelly KM Mayer RL Blonsky J. Treatment of primary headache in the emergency department. Headache. (2003) 43:1026–31. 10.1046/j.1526-4610.2003.03202.x 14629236

[12] Gupta MX Silberstein SD Young WB Hopkins M Lopez BL Samsa GP. Less is not more: underutilization of headache medications in a university hospital emergency department. Headache. (2007) 47:1125–33. 10.1111/j.1526-4610.2007.00846.x 17883517

[13] Kennis K Kernick D O'Flynn N. Diagnosis and management of headaches in young people and adults: NICE guideline. Br J Gen Pract. (2013) 63:443–5. 10.3399/bjgp13X670895 23972191 PMC3722827

[14] Bendtsen L Birk S Kasch H Aegidius K Sørensen PS Thomsen LL. Reference programme: diagnosis and treatment of headache disorders and facial pain. Danish Headache Society, 2nd Edition, 2012. J Headache Pain. (2012) 13(Suppl 1):S1–29. 10.1007/s10194-011-0402-9 22270537 PMC3266527

[15] De Luca GC Bartleson JD. When and how to investigate the patient with headache. Semin Neurol. (2010) 30:131–44. 10.1055/s-0030-1249221 20352583

[16] Ravishankar K. WHICH Headache to investigate, WHEN, and HOW? Headache. (2016) 56:1685–97. 10.1111/head.12998 27796030

[17] Friedman BW Serrano D Reed M Diamond M Lipton RB. Use of the emergency department for severe headache. A population-based study. Headache. (2009) 49:21–30. 10.1111/j.1526-4610.2008.01282.x 19040677 PMC2615458

[18] Knox J Chuni C Naqvi Z Crawford P Waring W. Presentations to an acute medical unit due to headache: a review of 306 consecutive cases. Acute Med. (2012) 11:144–9. 10.52964/AMJA.0563 22993744

[19] Do TP Remmers A Schytz HW Schankin C Nelson SE Obermann M. Red and orange flags for secondary headaches in clinical practice: SNNOOP10 list. Neurology. (2019) 92:134–44. 10.1212/WNL.0000000000006697 30587518 PMC6340385

[20] Granato A Morelli ME Cominotto F D'Acunto L Manganotti P. Adherence to guidelines of treatment of non-traumatic headache in the emergency department. Acta Neurol Belg. (2020) 120:19–24. 10.1007/s13760-020-01272-y 31965541

[21] Buckley NA Whyte IM Dawson AH Reith DA. Preformatted admission charts for poisoning admissions facilitate clinical assessment and research. Ann Emerg Med. (1999) 34:476–82. 10.1016/S0196-0644(99)80049-3 10499948

[22] Pohl H Do TP García-Azorín D Hansen JM Kristoffersen ES Nelson SE. Green Flags and headache: a concept study using the Delphi method. Headache. (2021) 61:300–9. 10.1111/head.14054 33405273

[23] Cortelli P Cevoli S Nonino F Baronciani D Magrini N Re G. Evidence-based diagnosis of nontraumatic headache in the emergency department: a consensus statement on four clinical scenarios. Headache. (2004) 44:587–95. 10.1111/j.1526-4610.2004.446007.x 15186303

